# Effect of Nasal Septum Deviation on the Choroidal Thickness

**DOI:** 10.7759/cureus.37840

**Published:** 2023-04-19

**Authors:** Ali A Yetkin, Mehmet Karataş

**Affiliations:** 1 Department of Ophthalmology, Adiyaman University Faculty of Medicine, Adiyaman, TUR; 2 Department of Otorhinolaryngology, Adiyaman University Faculty of Medicine, Adiyaman, TUR

**Keywords:** choroidal, nasal septum, optical coherence tomography, choroidal thickness, choroid

## Abstract

Introduction: Nasal cavity blood circulation and ocular blood circulation have common pathways for both arterial blood supply and venous drainage. Therefore, nasal pathologies can affect ocular blood circulation. This study aimed to evaluate the relationship between nasal obstruction and choroidal thickness.

Methods: A prospective study was planned by forming a group of 144 patients diagnosed with nasal septum deviation at the otorhinolaryngology clinic and 100 healthy voluntary individuals. Of the total, 69 patients with nasal right septum deviation were evaluated as Group 1, 75 patients with nasal left septum deviation as Group 2, and 100 healthy volunteers as the control group. Detailed ophthalmological examinations of all the participants were performed, and choroidal thickness was measured using spectral domain optical coherence tomography. The relationship between choroidal thickness and ocular parameters was evaluated and compared between the patient groups with nasal septum deviation and the control group.

Results: When the choroidal thickness measurements of the patients in Group 1 were examined, the choroidal thickness increased in all the regions in the eye contralateral to the deviation side (left), and intraocular pressure (IOP) was higher compared to the eye on the deviation side (right) and the control group at a statistically significant level. In Group 2, the choroidal thickness measurements increased in all the regions in the eye contralateral to the deviation side (right), and IOP was higher compared to the deviation side (left) and the control group.

Conclusions: We found that the patients with nasal septum deviation had higher choroidal thickness and IOP values in the eye contralateral to deviation.

## Introduction

Nasal congestion is a symptom frequently encountered by otolaryngologists and primary care physicians in clinical practice and can be caused by a wide variety of factors, such as anatomical, physiological, and pathophysiological [1]. Nasal septal deviation is among the most common causes of upper airway obstruction. Chronic upper airway obstruction resulting from this condition can lead to chronic hypoxia or hypercarbia due to alveolar hypoventilation. The obstruction may be chronic and severe and can even result in compensatory contralateral inferior turbinate hypertrophy. This can cause an increase in the surface area and blood supply of the inferior turbinate [2,3]. Nasal cavity blood circulation and ocular blood circulation have common pathways for both arterial blood supply and venous drainage [4]. The choroid is a vascular structure responsible for approximately 90% of the ocular blood flow, supplied by the posterior ciliary arteries. It plays a critical role in supplying oxygen and nutrients to the outer retina and in regulating the temperature of the retina through its blood flow. Due to its vascular nature, systemic conditions like diabetes, chronic heart failure, and hypertension can have an impact on the choroidal blood flow [3,5].

With the developments in enhanced depth imaging-optical coherence tomography (EDI-OCT) technology, the choroidal layer can now be evaluated in more detail, and changes in this layer can be detected more easily [6].

"Studies conducted in the past have indicated a potential link between abnormal choroidal thickness and either increased or decreased choroidal circulation" [7]. Therefore, this study aimed to evaluate the relationship between nasal obstruction and choroidal thickness.

## Materials and methods

After receiving institutional ethics committee approval, this study was conducted in accordance with the principles of the Declaration of Helsinki. Written informed consent was obtained from all the participants included in the study.

This prospective study included 144 patients who presented to the otorhinolaryngology polyclinic with nasal obstruction between September 2017 and September 2022 and were diagnosed with nasal septum deviation and 100 healthy individuals without nasal obstruction and any nasal discomfort or ocular pathology. All participants underwent a comprehensive otolaryngological examination including the oropharynx, nasal passages, nasopharynx, larynx, and hypopharynx. The examination was performed using a flexible fiberoptic endoscope. Of the total, 69 patients with nasal right septum deviation were evaluated as Group 1, 75 patients with left septum deviation as Group 2, and 100 healthy volunteers as the control group.

The detailed ophthalmological examinations of all the participants, including refractive error and visual acuity (VA) measurements, slit lamp examination, intraocular pressure (IOP) measurement with applanation tonometry, and dilated fundus examination were undertaken. The axial length (AL) of the eye was measured with an optical biometer (Lenstar LS 900; Haag Streit AG, Koeniz, Switzerland). Central corneal thickness (CCT) was measured from the pachymetry map obtained using a lens (CAM-L; 8 X 1024 A-scans) during EDI-OCT.

The study excluded individuals who had a refractive error of greater than ±1.0 D (diopter) in myopic, hyperopic, or astigmatic vision; those with ocular disorders such as glaucoma, diabetic retinopathy, or keratoconus; those who used contact lenses; those with a history of ocular surgery or trauma; those found to have vascular abnormalities during fundus examination; those with chronic systemic diseases, upper airway obstruction-related conditions (rhinitis, nasal polyposis, adenotonsillar hypertrophy, obstructive sleep apnea), autoimmune disease, chronic inflammatory disease, smoking, or long-term drug use; and those who could not undergo EDI-OCT or did not consent to the procedure.

The choroidal measurements of all the participants were performed using the choroidal mode of the EDI-OCT system (Heidelberg Engineering, Heidelberg-Germany) after the pupils were dilated with 1% cyclopentolate hydrochloride (Sikloplejin®, Abdi Ibrahim, Istanbul, Turkey). The measurements were undertaken in the morning to avoid diurnal variation. The choroidal measurement distance was taken as the hyperreflective line between the retinal epithelial line and the great vessel layers of the scleral interface. The choroid was measured in the foveal center (FC) in the horizontal nasal and temporal quadrants at 500 μm intervals, at seven points up to 1500 μm distances (nasal 1500 μm, nasal 1000 μm, nasal 500 μm, subfoveal, temporal 500 μm, temporal 1000 μm, and temporal 1500 μm). An experienced physician performed the measurements manually.

Statistical analysis

Statistical analysis was performed using the Statistical Package for the Social Sciences, version 25.0 (SPSS Inc., Chicago, IL). Data were expressed as mean and standard deviation. The normality of numerical data distribution was evaluated with the Kolmogorov-Smirnov test. The patient and control groups were evaluated with the independent parametric two-samples t-test or non-parametric Mann-Whitney U test, and the two eyes of the patients were compared using the parametric dependent-samples t-test or non-parametric Wilcoxon test. A P value of <0.05 was considered statistically significant.

## Results

Group 1 consisted of 69 patients, of whom 34 were male and 35 were female, with the age range being 28.0±8 years. In Group 2, there were 75 patients (38 male and 37 female), and the age range was 30.0±9 years. In the voluntary control group, 48 of the 100 patients were male, and the age range was 29.6±8 years.

The ocular parameters and choroidal thickness measurements of the patients in Group 1 are shown in Table 1.

**Table 1:** Ocular parameters and choroidal thickness measurements of the patients in Group 1 P<0.05. *Comparison of the eye contralateral to the deviation side (left) with the eye on the deviation side (right); ^†^Comparison of the eye contralateral to the deviation side (left) with the control group. IOP, intraocular pressure; CCT, central corneal thickness; AL, axial length; VA, visual acuity; FC, foveal center

	Right eye	Left eye	Control	P*	P^†^
IOP	13.23±1.296	15.51±1.244	13.34±1.719	0.000	0.000
CCT	530.01±29.002	529.33±29.274	532.68±21.480	0.438	0.140
AL	23.03±1.014	23.01±1.050	23.00±1.025	0.739	0.876
VA	9.768±0.4584	9.812±0.3939	9.79±0.478	0.257	0.964
Temp 500	323.25±63.393	352.03±68.226	319.02±62.267	0.000	0.001
Temp 1000	311.25±63.264	336.72±66.827	308.47±63.574	0.000	.003
Temp 1500	298.49±64.982	320.94±67.588	294.28±56.169	0.000	0.006
FC	340.46±64.016	371.13±71.685	338.77±59.183	0.000	0.002
Nasal 500	327.45±65.443	354.35±69.595	324.80±69.549	0.000	0.007
Nasal 1000	314.32±65.620	338.71±67.397	311.37±67.281	0.000	0.010
Nasal 1500	295.43±65.405	322.74±66.382	291.56±66.936	0.000	0.003

There was no significant difference in the ocular parameters of CCT, AL, and VA in the comparison of the two eyes in Group 1 and the comparison between the patients and controls (p>0.05 and p>0.05, respectively). However, we determined that the IOP value of the patients in the eye contralateral to the deviation side (left) was higher compared to the eye on the deviation side (right) and the control group at a statistically significant level (p<0.05 and p<0.05, respectively).

When the choroidal thickness measurements of the patients in Group 1 were examined, it was observed that the choroidal thickness increased in all the regions in the eye contralateral to the deviation side (left), and IOP was higher in this eye compared to the deviation side (right) and the control group at a statistically significant level (p<0.05 and p<0.05, respectively).

The ocular parameters and choroidal thickness measurements of the patients in Group 2 are shown in Table 2.

**Table 2:** Ocular parameters and choroidal thickness measurements of the patients in Group 2 P<0.05. *Comparison of the eye contralateral to the deviation side (right) with the eye on the deviation side (left); ^†^Comparison of the eye contralateral to the deviation side (right) with the control group. IOP, intraocular pressure; CCT, central corneal thickness; AL, axial length; VA, visual acuity; FC, foveal center

	Right eye	Left eye	Control	P*	P^_†_^
IOP	15.69±1.355	13.55±1.266	13.34±1.719	0.000	0.000
CCT	532.83±22.450	533.51±24.017	532.68±21.480	0.676	0.866
AL	22.96±0.979	23.00±1.027	23.00±1.025	0.475	0.783
VA	9.76±0.489	9.80±0.465	9.79±0.478	0.405	0.602
Temp 500	348.01±67.786	320.05±66.522	319.02±62.267	0.000	0.003
Temp 1000	332.13±67.533	305.33±66.246	308.47±63.574	0.000	0.012
Temp 1500	313.84±65.643	290.81±64.374	294.28±56.169	0.000	0.040
FC	368.19±71.163	336.63±66.751	338.77±59.183	0.000	0.005
Nasal 500	351.91±69.289	322.35±66.131	324.80±69.549	0.000	0.016
Nasal 1000	335.15±67.284	308.44±65.040	311.37±67.281	0.000	0.030
Nasal 1500	316.67±64.211	293.05±63.314	291.56±66.936	0.000	0.017

The ocular parameters (CCT, AL, and VA)​​ of the patients in Group 2 did not statistically significantly differ when compared between the eyes and with the control group. However, we observed that the IOP value of the eye contralateral to the deviation side (right) was higher compared to the deviation side (left) and the control group, and this was statistically significant (p<0.05 and p<0.05, respectively).

The choroidal thickness measurements of the patients in Group 2 revealed that the choroidal thickness increased in all the regions in the eye contralateral to the deviation side (right), and IOP was higher on this side compared to the deviation side (left) and the control group at a statistically significant level (p<0.05 and p<0.05, respectively).

## Discussion

The choroid layer is the thickest part of the macular region and is a rich vascular structure with the greatest blood flow in the human body [8]. One of the most frequent causes of upper airway obstruction is nasal septal deviation. If the deviation results in chronic obstruction of the upper airway, it can cause chronic hypoxia, which may affect blood flow to the choroid. The choroid layer provides the majority of ocular blood flow and is involved in the feeding, oxygenation, temperature regulation, and metabolic activity of the retinal pigment [5]. Today, the choroidal layer can be better evaluated with the development of EDI-OCT technology, and changes that may occur in the choroid can be better visualized in diseases that can affect the choroidal layer, such as central serous retinopathy, polypoidal choroidal vasculopathy, and Vogt-Koyanagi-Harada disease [6,7,9,10].

In investigating the effect of chronic heart failure on choroidal thickness, Altinkaynak et al. found that the choroidal layer was thinner, which they attributed to the decrease in choroidal blood circulation due to heart failure [9]. In evaluating choroidal thickness in patients with cardiovascular disease, Yeung et al. reported that choroidal thickness decreased in coronary artery disease and carotid artery stenosis, and this was caused by decreased blood flow [10]. Similarly, Karalezli et al. investigated the effect of sleep apnea syndrome on the choroid and determined that the choroidal layer was thinner in patients with this disorder, and this was probably due to reduced choroidal blood flow [11].

Uzlu et al. reported an increase in choroidal measurements in patients with thyroid-related ophthalmopathy and suggested that this might be a result of increased choroidal blood flow by venous obstruction [12]. In a study examining choroidal changes in patients with Graves’ disease, Bruscolini et al. found that the decrease in choroidal drainage in the ophthalmic veins caused the choroidal layer to thicken [13]. The authors explained this with increased choroidal thickness due to increased hydrostatic pressure toward the ophthalmic vein, resulting in the enlargement of the veins in the choroidal layer and thus choroidal blood volume [14].

In an animal study, Zhang et al. found that choroidal thickness increased as choroidal blood flow increased [15]. Bezgin et al. reported that there was no correlation between nasal septum deviation and choroid thickness in their study [3]. However, in a survey, Şahin et al. compared choroidal thickness before and after septoplasty in patients with a deviated septum. As a result of this study, they reported a significant correlation between nasal septum deviation and choroidal thickness and that choroidal thickness increased significantly after surgical treatment of these patients compared to preoperative values [16]. Consistent with the studies in the literature, we found an increase in all seven regions of the choroidal layer, from which we performed the choroid layer measurements in the eyes contralateral to septum deviation among the patients in Group 1 and Group 2 when compared with the eyes on the other side and the control group. A possible mechanism for this is nasal septum deviation causing compensatory contralateral inferior turbinate hypertrophy. As a result of this hypertrophy, increased surface area leads to an increase in blood circulation [2,17,18]. This increase in blood circulation then results in an increase in blood circulation in the choroid tissue through the common pathways of ocular and nasal cavity blood circulation in terms of both arterial blood supply and venous drainage. We consider that this increased blood circulation causes the thickening of the choroidal layer [1,19].

In studies exploring the relationship between choroidal layer changes and ocular parameters, the thinning of the choroidal layer has been correlated with advanced age, increased myopia, and increased AL, while choroidal thickening has been correlated with increased IOP, corneal thinning, and amblyopia [20-26]. In our study, we did not detect a significant difference in the ocular parameters of VA, AL, and CCT of the patients between Group 1, Group 2, and the control group. However, in Group 1 and Group 2, there was a significant increase in IOP in the eye contralateral to septum deviation when compared with the eye on the other side and the control group. On examining IOP and choroidal changes as a result of postural changes in patients with multiple system atrophy and Parkinson’s disease, De Bernardo et al. attributed the increase in IOP to the thickening of the choroidal layer as a result of increased choroidal blood flow and thickened choroidal layer [23]. Shinojima et al. found that the subfoveal choroidal thickness increased in patients during head-down tilt. Increased choroidal thickness also affects the sclera; however, the sclera cannot expand since it consists of a hard and thick layer. Therefore, the authors suggested that this caused an increase in IOP due to increased episcleral venous pressure [14]. Similarly, we consider that the increase in IOP in the eye contralateral to septum deviation in our study was due to the thickening of the choroidal layer.

## Conclusions

In this study, we found that the patients in Group 1 and Group 2 had higher choroidal thickness and IOP values in the eyes contralateral to septum deviation. According to these findings, we recommend examining the choroidal layer and performing IOP measurements in patients with nasal septum deviation and screening patients with high IOP values for nasal septum deviation. However, there is a need for further comprehensive studies on the relationship between nasal septum deviation and choroidal changes to support our findings.
